# Outbreak of leptospirosis among triathlon participants in Germany, 2006

**DOI:** 10.1186/1471-2334-10-91

**Published:** 2010-04-10

**Authors:** Stefan Brockmann, Isolde Piechotowski, Oswinde Bock-Hensley, Christian Winter, Rainer Oehme, Stefan Zimmermann, Katrin Hartelt, Enno Luge, Karsten Nöckler, Thomas Schneider, Klaus Stark, Andreas Jansen

**Affiliations:** 1 Baden-Württemberg State Health Office, District Government Stuttgart, Germany; 2 Gesundheitsamt Rhein-Neckar-Kreis, Heidelberg, Germany; 3 Postgraduate Training of Applied Epidemiology (PAE), Berlin, Germany; 4 Karl-Ruprecht University Heidelberg, Department of Hygiene, Heidelberg, Germany; 5 Federal Institute for Risk Assessment (BfR), Berlin, Germany; 6 Medical Clinic I, Campus Benjamin Franklin, Charité, Berlin, Germany; 7 Department for Infectious Disease Epidemiology, Robert Koch Institute, Berlin, Germany

## Abstract

**Background:**

In August 2006, a case of leptospirosis occurred in an athlete after a triathlon held around Heidelberg and in the Neckar river. In order to study a possible outbreak and to determine risk factors for infection an epidemiological investigation was performed.

**Methods:**

Participants of the triathlon were contacted by e-mail and were asked to fill out a standardized questionnaire. In addition, they were asked to supply a serum sample for laboratory diagnosis of leptospirosis. A confirmed case patient was defined as a clinical case (i.e. fever and at least one additional symptom suggestive for leptospirosis) with at least two of the following tests positive: ELISA IgM, latex agglutination testing, or microscopic agglutination testing. Rainfall and temperature records were obtained.

**Results:**

A total of 142 of 507 triathletes were contacted; among these, five confirmed leptospirosis cases were found. Open wounds were identified as the only significant risk factor for illness (p = 0.02). Heavy rains that preceded the swimming event likely increased leptospiral contamination of the Neckar River.

**Discussion:**

This is the first outbreak of leptospirosis related to a competitive sports event in Germany. Among people with contact to freshwater, the risk of contracting leptospirosis should be considered by health care providers also in temperate countries, particularly in the summer after heavy rains.

## Background

Leptospirosis is a zoonotic disease caused by spirochaetes of the genus *Leptospira*. Transmission to humans results from exposure to urine of infected animals, either by direct contact or - more frequently - through contaminated soil or water [1]. Clinical manifestations range from mild, flu-like symptoms to life threatening disease characterized by jaundice, renal impairment and hemorrhage [2].

Leptospirosis has recently been classified as a reemerging infectious disease, particularly in tropical and subtropical regions [2,3]. In Europe, leptospirosis was historically associated with agricultural exposure risks [4-7]. While some occupational exposures continue to exist, exposures related to travelling and recreational activities have emerged as an important route of transmission in recent years [8].

In August 2006 a case of leptospirosis was detected in the federal state of Baden-Württemberg by routine surveillance. A 43-years old female was referred to a hospital with high fever, and subsequently developed renal failure and hepatitis. Explorative assessment of possible risk factors by the local health authorities revealed swimming in the Neckar River in Heidelberg during a triathlon three days before onset of symptoms as the most likely source of infection. In order to determine the extent of a possible outbreak and possible risk factors for infection, an outbreak investigation was performed.

## Methods

In September 2006, we conducted a retrospective cohort study among the triathletes officially registered for the event. The participants were contacted by e-mail, and were asked to fill out a standardized questionnaire. A written consent was obtained from each participant of the outbreak investigation. The questionnaire included information on demographics, work and travel history, and clinical information. In addition, triathletes were asked to provide a serum sample for the investigation of antibodies against leptospires. A case patient was defined as an individual who participated in the triathlon in Heidelberg on August 6^th ^2006, and who had fever and at least one other symptom suggestive of leptospirosis (i.e. renal impairment, meningitis, headache, flu-like symptoms, vomiting) [9] within two days to six weeks after the event, as well as serological test results positive for antibodies against leptospires in at least two of three test assays. Samples were screened using the Biosave Leptospira Latextest (BIOS, Munich, Germany). The latex particles of this assay are coated with antigens of *L. grippotyphosa*. Two serum dilutions (1:2 and 1:10) were tested. Only clearly visible agglutinations occurring within five minutes were read as positive results. All samples identified as positive by latex agglutination were confirmed with use of a commercial IgM ELISA kit (Virion/Serion, Würzburg, Germany) [10]. Samples were further tested for antibodies using the microagglutination test (MAT). Seventeen reference strains comprising 14 serogroups and 17 serovars (serovars Australis, Autumnalis, Bataviae, Bratislava, Canicola, Copenhageni, Grippotyphosa, Hardjo, Pomona, Saxkoebing, Sejroe, Tarassovi, Ballum, Icterohaemorrhagiae, Pyrogenes, Hebdomadis and Javanica) were used for MAT. Antibody titres ≥1:100 were considered positive for leptospirosis. The association between exposures and leptospirosis outcome were examined by univariate analysis. Risk ratios (RR) and their 95% confidence intervals (95% CI) and p values were calculated. Using Fisher's exact test, variables at p < 0.05 were considered significant. Analyses were done with SPSS 15 (SPSS Inc., Chicago, USA). Rainfall and ambient temperature records were obtained from the German meteorological office.

This epidemiological study was performed in compliance with the Helsinki Declaration. It was conducted within the framework of the German Protection against Infection Act (Infektionsschutzgesetz). Mandatory regulations were observed.

The study was performed in accordance with the standards for data protection established at the Robert Koch Institute, Germany.

## Results

A total of 507 participants swam the 1.7 km distance in the Neckar river on August 6^th^. Of these, 142 (28%; 82% males) responded to the e-mail and submitted a serum sample. Five participants (4%) met the definition for a leptospirosis case (3 males). Mean age was 35 years (SD 8) in case patients, and 37 years (SD 8) in healthy participants (n.s.). The median incubation period was 15 days (range: 2-34).

Of the 142 sera tested, 6 (4%) were positive for IgM antibodies, and 5 of these were positive by latex agglutination testing. MAT was found positive in one of these five patients. In this patient, antibodies were found against several serogroups (Australis 1:200; Bataviae 1:800; Canicola 1:400; Copenhageni 1:400; Sejroe 1:800; Ballum 1:100; Icterohaemorrhagiae 1:100; Pyrogenes 1:800). In one participant without clinical symptoms, seropositivity for serogroups Icterohaemorrhagiae/Copenhageni 1:100 was found.

Clinical disease was mild in most cases with fever most frequently reported (100%) followed by headache (4/5) and muscle pain (4/5). Diarrhea and renal impairment were reported from single patients, respectively. Jaundice, pulmonary haemorrhage, meningoencephalitis, or other severe manifestations of leptospirosis were not reported. No deaths were reported. Three of the 5 patients required hospitalization.

In univariate analysis, having wounds was the only significant risk factor (p = 0.02) (Table 1). Swallowing water, swim time, or having worn goggles while swimming were not associated with illness.

**Table 1:** Risk factors for developing leptospirosis (univariate analysis).

Risk	Case patients exposed n (%)	Healthy individuals n (%)	RR	p-value
Wounds	3 (100)	20 (25)	n.d.	0.02
Swallowing Water	3 (100)	100 (95)	n.d.	0.7
Eye Protection	5 (100)	122 (90)	n.d.	1
Traveling	2 (40)	58 (44)	0.9	1
Gardening	0 (0)	31 (23)	n.d.	0.6
Farming	0 (0)	7 (5)	n.d.	1
Contact to animals	1 (20)	5 (4)	5.5	0.2
Male sex	3 (60)	113 (84)	0.3	0.2
Previous freshwater contact	3 (75)	77 (58)	2.2	0.6

n.d.: not defined.

**Figure 1:**
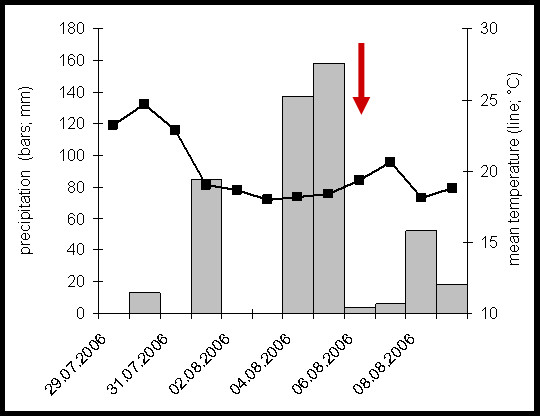
**Rainfall and temperature records for Heidelberg around the time of the triathlon (6^th ^August, 2006)**. The date of the triathlon is indicated by the red arrow.

The area where the outbreak took place recorded an average monthly temperature of 25.2°C in July and 17.8°C in August 2006. Between 4^th ^and 5^th ^August (the two days before outbreak onset), the average daily temperature ranged between 18.2-18.4°C. During this time, a period of heavy precipitation was recorded when rainfall was 148 mm per day on average, which corresponds to 148 liters of rainwater per square meter (Figure 1).

## Discussion and Conclusion

Our study describes the first documented outbreak of leptospirosis related to a competitive water sports event in Germany. Although sporadic cases of leptospirosis related to recreational freshwater exposure have been described before, leptospirosis outbreaks typically occurred in more tropical countries. In temperate regions, direct contact to infected reservoir animals (i.e. husbandry, farming) was considered as the traditional route of infection so far. This outbreak might be an indicator for an ongoing change in the epidemiology of leptospirosis towards becoming a more widespread environmental hazard.

Conditions that favor the environmental spread of leptospirosis largely depend upon climate conditions. Consequently, leptospirosis has recently been identified as a climate-sensitive disease [11,12]. Results of current climate research predict that temperatures will continue to rise in the future resulting in more periods of very warm weather [13]. In addition, periods of sudden heavy rain are likely to become more common in Europe [14]. Like in the present outbreak, dry periods of little or no rain, followed by days of heavy rain seem to be a perfect setting for leptospirosis epidemics. Similar conditions were also observed during a triathlon in Springfield, USA, resulting in more than 50 leptospirosis cases [15]. With Europe and other regions moving towards warmer winters and more tropical summers, a greater number of leptospirosis cases and outbreaks will likely occur in the future.

A disrupted skin barrier is the classically acknowledged route of transmission, and we found that the presence of wounds before swimming was associated with the infection. Open wounds likely facilitate the entry of leptospires directly into the bloodstream, thereby increasing the quantity of pathogens that enter the body in a given period of exposure. Our findings suggest that protective clothing and/or covering lesions with waterproof dressings may decrease the risk of leptospirosis in individuals exposed to potentially contaminated water. Due to the high temperature of the river water during the sports event (>23°C), athletes were not allowed to wear a wet suit. In the Springfield outbreak swallowing water was identified as the predominant risk factor for infection. We did not find a similar association in the Heidelberg outbreak, perhaps due to a lower grade of contamination, a shorter swim time, or high exposure prevalence also in healthy individuals.

The clinical presentation of leptospirosis is relatively unspecific, and it is easily mistaken for influenza-like illness or similar diseases. One participant of the Heidelberg event with a positive serology for leptospirosis was hospitalized because of a severe febrile infection which he experienced after a triathlon the year before. He was discharged, however, without a diagnosis being made. Failure to diagnose leptospirosis bears implications for disease progression and emphasizes the importance of improving knowledge among general practitioners and hospital staff to facilitate early recognition and treatment.

A limitation of our study is the absence of environmental and animal samples. Thus, detection of leptospires in water samples by culture or another suitable method could not prove their presence at the questionable time and place of exposure. Due to a relatively low sensitivity of such environmental investigations, however, failure to find the agent does not necessarily mean its absence. Thus, screening large bodies of freshwater for leptospires should not guide public health authorities in making decisions regarding the safe recreational use of water.

A possible way of assessing the likelihood of contamination is to check the area around the water body for the presence of animals that may act as infection sources. In the present outbreak, however, the event took place in a river, and we were not able do determine the possible area of interest for screening relevant reservoir animals.

In similar investigations, rodents were identified as reservoirs and source of human infections [9,16]. Interestingly, we detected anti-leptospira antibodies against serogroups Icterohaemorrhagiae/Copenhageni in one of our cases by MAT. These serogroups are frequently found in rodents, namely rats. Although serogroups identification by MAT has to be interpreted cautiously [17], we suggest that excretion of leptospires by rodents near the river area probably caused this outbreak.

The limitation to obtain MAT confirmation in all specimens that tested positive by ELISA may be explained by a relatively low sensitivity but high specificity of this agglutination test. Unfortunately, it was not possible to isolate the pathogen and type leptospiral serovars, because the outbreak investigation and the sera collection commenced a month after the triathlon, and isolation of the leptospires was not attempted by the physicians of the three hospitalized cases.

To conclude, our study advices that individuals who conduct water sports should be informed of the potential risk of leptospirosis. This should be done in the light of unspecific symptoms which may occur during leptospirosis. If an illness compatible with leptospirosis develops, early diagnosis accompanied by immediate medical help and information of the health care provider about the exposure are required. Swimming with wounds or after heavy rains represents a particular risk for contracting this potentially live-threatening infection.

## Competing interests

The authors declare that they have no competing interests.

## Authors' contributions

SB, IP, OBH, CW, RO, KH, TS, KS, and AJ participated in the design of the study and performed the epidemiological investigation. SB and AJ were responsible for data analysis.

KN, EL, and ST carried out the microbiological investigations. All authors read and approved the final manuscript.

## Pre-publication history

The pre-publication history for this paper can be accessed here:

http://www.biomedcentral.com/1471-2334/10/91/prepub
